# Prevalence of frontotemporal dementia in community-based studies in Latin America: a systematic review

**DOI:** 10.1590/S1980-57642013DN70100005

**Published:** 2013

**Authors:** Nilton Custodio, Eder Herrera-Perez, David Lira, Rosa Montesinos, Liliana Bendezu

**Affiliations:** 1 Department of Neurology, Clinica Internacional, Lima, Peru. Unit of Cognitive Impairment and Dementia Prevention, Clinica Internacional, Lima, Peru.; 2 Unit of Cognitive Impairment and Dementia Prevention, Clinica Internacional, Lima, Peru. Research Projects Unit, National Institute of Child Health. Lima, Peru.; 3 Unit of Cognitive Impairment and Dementia Prevention, Clinica Internacional, Lima, Peru. Rehabilitation Medicine Unit, Clinica Internacional, Lima, Peru.

**Keywords:** prevalence, frontotemporal lobar degeneration, frontotemporal dementia, primary progressive nonfluent aphasia

## Abstract

Latin America (LA) is experiencing a rise in the elderly population and a consequent increase in geriatric problems such as dementia. There are few epidemiological studies assessing the magnitude of dementia and dementia subtypes in LA.

**OBJECTIVE:**

To identify published community-based studies on the prevalence of FTD in LA countries.

**METHODS:**

A database search for door-to-door studies on FTD prevalence in LA was performed. The search was carried out on Medline, Embase, and LILACS databases for research conducted between 1994 and 2012. The main inclusion criteria were: use of any internationally accepted diagnostic criteria and investigation of community samples.

**RESULTS:**

Four hundred and ninety two articles were found, of which 26 were initially pre-selected by title or abstract review. Five studies from 3 different countries were included. The FTD prevalence rates in community-dwelling elderly were 1.2 (Venezuela), 1.3 (Peru) and 1.7-1.8 (Brazil) per thousand persons, depending on age group.

**CONCLUSION:**

The FTD prevalence in LA studies showed values mid-way between those observed in western and in oriental populations. Despite the magnitude of this problem, epidemiological information on FTD remains scarce in LA.

## Abstract (pt)

A América Latina (AL) está experimentando um aumento na população de idosos e um consequente aumento nos problemas geriátricos, como demência. Existem poucos estudos epidemiológicos avaliando a magnitude de demência e demência subtipos na AL

**OBJETIVO:**

Identificar publicações baseadas em estudos sobre a prevalência da FTD em países da AL.

**MÉTODOS:**

A pesquisa realizada foi por estudos de prevalência de FTD em comunidade na AL. A pesquisa foi realizada em Med-line, Embase, e LILACS no período entre 1994 e 2012. Os principais critérios de inclusão foram: utilização de quaisquer critérios internacionalmente aceitos de diagnóstico e investigação de amostras em comunidade.

**RESULTADOS:**

Quatrocentos e noventa e dois artigos foram encontrados, dos quais 26 foram inicialmente pré-selecionados pelo título ou fiscalização do abstract. Cinco estudos de 3 países diferentes foram incluídos. As taxas de prevalência na comunidade em idosos com FTD eram 1,2 (Venezuela), 1,3 (Peru) e 1,7-1,8 (Brasil) por mil pessoas, dependendo da faixa etária.

**CONCLUSÃO:**

A prevalência FTD em estudos da AL, apresentaram valores intermediários entre os observados em populações ocidentais e orientais. Apesar da magnitude do problema, informações epidemiológicas sobre FTD permanecem escassas em AL.

## INTRODUCTION

Latin America (LA) is experiencing a rise in the elderly population as lifespan increases, ^1^ leading to a rise in prevalence of chronic medical and geriatric conditions, including dementia. For these reasons, dementia is emerging as an important public health problem^2^ and a major cause of disability and mortality in this region.^3^

To date, few epidemiological studies assessing the magnitude of dementia^2^ have been conducted in LA, where current prevalence estimates vary widely.^4^ However, a recent review reported an overall prevalence of dementia of 7.1% in elderly aged 65 years and over from six LA countries. Alzheimer's disease and vascular dementia were the most frequent causes of dementia.^5^ No reviews are available on frontotemporal dementia (FTD) prevalence in LA.

FTD is a clinically and pathologically heterogeneous syndrome, characterized by progressive decline in behavior or language functions associated with frontal and anterior temporal lobe degeneration^6-8^ and non- Alzheimer pathology (9,10). FTD accounts for 5-6% of all dementias and three-quarters of cases can present among patients under 65, with FTD being considered an early-onset dementia.^7,11,12^ However, some recent studies have shown that FTD may be more common than previously thought.^13^

The main objective of this collaborative study was to analyze data from community-based studies on the prevalence of FTD in LA countries and to verify whether the LA prevalence differs from rates reported in other regions of the world.

## METHODS

**Definition of terms.** The first statement of consensus on the diagnostic criteria for FTD was published in 1994 (Lund-Manchester research criteria, LMRC), differentiating clinical and neuro-pathological diagnostic features. The LMRC include three FTD symptom constellations: [1] behavioral symptoms; [2] affective symptoms; and [3] speech disorder. The onset has to be insidious and the course invariably progressive. The criteria do not describe in detail the required severity of the symptoms, or how many symptoms or symptom constellations have to be present for a diagnosis. Three distinct neuro-pathological types can be distinguished: [1] frontal lobe degeneration type; [2] Pick-type; and [3] motor neuron disease type.^14^

At a later date, publication of consensus criteria by Neary and colleagues occurred. Under the nomenclature used in the Neary diagnostic criteria, the term FTLD encompasses three distinct clinical variants that can be distinguished based on the early and predominant symptoms: a behavioral-variant (bvFTLD) and two language variants (semantic dementia and progressive nonfluent aphasia).^15^ Each syndrome has a unique anatomy: bvFTLD is characterized by bifrontal atrophy (frontal-variant FTLD, fvFTLD), semantic dementia (SD) by anterior temporal atrophy (temporal-variant FTLD, tvFTLD), and progressive nonfluent aphasia (PNFA) by left peri-sylvian atrophy.^8,16^ While one clinical syndrome tends to predominate early on, with time atrophy tends to spread to previously unaffected brain regions involving the frontal and temporal lobes more diffusely, and the clinical syndromes may overlap.^17^

A recent study has proposed a new classification system which establishes four different syndrome subdivisions: [1] a frontal or behavioral variant (fvFTLD); [2] SD or Semantic variant Progressive Primary Aphasia (PPA-semantic); [3] PNFA or Nonfluent/agrammatic variant PPA (PPA-agrammatic); and [4] logopenic progressive aphasia or Logopenic variant PPA (PPA-logopenic). These variants differ in their clinical presentation, cognitive deficits, and affected brain regions.^18,19^

**Study search.** Studies published between 1994 and 2012 were retrieved from the following databases: Medline, Biomed Central, Embase, Scopus, Scirus, PsychInFO, LILACS and IBECS. Ovid, Ebsco, Proquest, Cochrane Library, Cochrane Library Plus, and the WHO/PAHO databases were also searched. All languages were considered. The authors independently searched for the terms ("Frontotemporal Lobar Degeneration" OR "Frontotemporal Dementia" OR "Primary Progressive Nonfluent Aphasia" OR "Semantic dementia" OR "Pick Disease of the Brain" OR "Logopenic") AND ("Prevalence" OR "Frequency" OR "Epidemiology" OR "Survey" OR "Community-based" OR "Cross-sectional") AND ("Latin America" OR each of the 20 Latin American country names). Since we were aware of a few investigations on the specific prevalence of FTD that had been published we also searched for global dementia studies. Abstracts of articles in any language were independently reviewed by the authors. The only inclusion criterion was that the study should be population-based. A secondary search of reference lists of the identified studies was also conducted. The latest date of publication for inclusion of articles in the study was the 30^th^ of November 2012.

**Study selection.** Studies were selected based on the following inclusion criteria: [1] original articles that estimated FTD prevalence in community samples using any previously indicated internationally accepted diagnostic criteria; [2] original articles that estimated dementia prevalence in community samples using any internationally accepted diagnostic criteria (DSM-III-R, DSMIV, DSM-IV-TR, ICD-9 or ICD-10) which reported the prevalence of dementia subtypes; [3] available from any bibliographic or academic database.

**Characterization of studies evaluated.** The following variables were recorded for each study: authors, publication year, site of study, type of study, age range studied, sample composition (inclusion of institutionalized subjects, urban or rural provenance), total sample size, sample design, loss of subjects, sub-sample size by age group and gender, FTD prevalence within age range studied and at 5-year intervals, prevalence in each gender and age interval, diagnostic criteria, use of computed tomography and laboratory exams.

**Ethics.** No approval from the ethics committee was required since the study was carried out solely with data published in the world literature.

## RESULTS

**Studies selected.** A total of 492 articles were retrieved, from which 26 were initially pre-selected by title or abstracts review. Two articles could not be accessed and another was a poster presentation. Two articles were studies in hospital settings. One article only showed incidence data. Ten articles only showed overall dementia prevalence data. Ultimately, ten articles showed dementia data by subtype and were finally reviewed.

Eight studies carried out in four LA countries were described in these ten articles (two studies were partially described in two articles each). The countries were the following: Brazil (Yamada et al. 2002, Herrera et al. 2002, Lopes 2010, Bottino 2007, Bottino et al. 2008, Scazufca et al. 2008), Cuba (Llibre et al. 1999), Venezuela (Maestre et al. 2002, Molero et al. 2007), and Peru (Custodio et al. 2007).

The Bottino, Scazufca, and Llibre studies presented dementia data by subtype but did not show the FTD diagnostic criteria where used. The Yamada study was limited to the Japanese immigrant population. Therefore, these studies were not included, giving a final total of four studies from three countries (Table 1).

**Table 1:** Characteristics of the four population-based studies included in the present review.

Author and reference	Site of study	Setting	Type of study	Age group	Sample size		Sample design	Diagnostic criteria	
					**Expected**	**Effective**		**Overall dementia**	**FTD**
Herrera, et al. 2002^34^	Catanduva, São Paulo, Brazil	Urban	Survey, triphasic	≥65 years	1700	1656	Random sampling: systematic	DSM-IV	Lund criteria
Maestre, et al. 2002^35^ Molero, et al. 2007^36^	Santa Lucia, Maracaibo, Venezuela	Urban	Survey, triphasic	≥55 years	3756	2438	Whole population	DSM-IV	Neary criteria
Lopes, 2010^37^	Ribeirão Preto, São Paulo, Brazil	Urban*	Survey, biphasic	≥60 years	1110	1145	Random sampling: systematic and stratified	DSM-IV and ICD-10	Lund criteria
Custodio, et al. 2007^38^	Cercado de Lima, Lima, Peru	Urban	Survey, triphasic	≥65 years	2958	1532	Random sampling: systematic	DSM-IV	Neary criteria

FTD: frontotemporal dementia; DSM-IV: Diagnostic and Statistical Manual of Mental Disorders, version IV; ICD-10: International Classification of Diseases, 10^th^ edition;

* Setting unreported (assumed by site features).There were no restrictions for socioeconomic or educational status.

All studies were carried out over more than one phase. The first phase included at least a socio-demographic and health questionnaire, a cognitive (Mini-Mental State Examination or Short Portable Mental Status Questionnaire) and functional (Pfeffer Functional Activities Questionnaire, Lawton and Brody Instrumental Scale or Bayer-Activities for Daily Living / Activities for Daily Living- International Scale) evaluations. All subjects with scores indicating dementia were considered to have suspected dementia and selected for phase II of the study. In phase II, selected subjects were submitted to clinical, neurologic, and cognitive evaluations with the same tests and other more complex cognitive tests. Subjects that fulfilled the Diagnostic and Statistical Manual of Mental Disorders (4^th^ edition) diagnostic criteria for dementia were selected for phase III.

The last phase included a clinical examination (at least one neuropsychological test) and laboratory and imagenological evaluation to rule out secondary causes of dementia. Based on the data from all phases, the clinical diagnoses were reached on a consensual basis, according to previously published standardized criteria for each illness. In the Lima study, the neuropsychological testing was conducted during the second phase. In the Ribeirão Preto study phases II and III were executed together. None of the studies provided information on the validity of each of the tests used in each phase of the investigation.

**Prevalence of dementia and FTD in community-based studies.** The results of LA studies estimating prevalence of all dementia in individuals 65 years and older, were as follows: Brazil 7.1%-7.2%; Venezuela: 13.3%; Peru: 6.9%. All studies were conducted in urban settings, without restrictions for educational or socioeconomic levels in the subject selection process, and diagnosed three cases of FTD (except the Lopes study, which found two cases). Of patients with overall dementia, between 1.5% and 2.8% had FTD (Table 2).

**Table 2:** Prevalence of frontotemporal dementia and overall dementia in four Latin American studies.

Country of study	Site of study	Age group	Prevalence				Cause of dementia	
			**Overall dementia (95% CI)**	FTD*	UC*		**FTD (%)**	**UC (%)**
Brazil^34,37^	Catanduva, São Paulo	≥65	7.1% (6.0-8.5)	0.18%	0.90%		2.60	12.70
	Ribeirão Preto, São Paulo	≥60	6.0% (4.6-7.3)	0.17%	0.43%		2.80	7.20
		≥65	7.2% (5.7-8.6)					
Venezuela^35,36^	Santa Lucia, Maracaibo	≥55	8.04% (7.01-9.19)	0.12%	0.45%		1.53	5.61
		≥65	13.27%*					
Peru^38^	Cercado de Lima, Lima	≥65	6.85% (5.53-8.08)**	0.13%	0.87%		1.90	12.70

FTD: frontotemporal dementia; UC: undetermined cause of dementia;

* Data calculated by authors;

** Data on confidence interval provided by author. Only crude non-adjusted data given.

In the Brazilian studies, the authors described very similar rates of overall dementia prevalence for elderly aged 65 years and older. Both studies were conducted in Portuguese speaker populations from São Paulo using the same diagnostic criteria for dementia and FTD. The Catanduva study was implemented in three phases and the clinical diagnosis was reached by three neurologists. The Ribeirão Preto study however, was conducted in two phases and the clinical diagnosis was reached by a medical team consisting of psychiatrists, a neurologist and a geriatrician. However, in the Catanduva survey nursing home residents were also included.

Highest and lowest overall dementia prevalence in the elderly populations aged 65 years and older was observed in Venezuelan and Peruvian studies, respectively. Both studies were conducted in Spanish-speaking populations, spanned three phases, and used the same diagnostic criteria for overall dementia and FTD. The proportion of subjects that did not complete the study was over 30% in the Venezuelan survey and 40% in the Peruvian survey.

In the LA dementia prevalence studies included in the review, the proportion of demented with an undetermined cause of dementia had the same range of variation among Spanish-speaking populations and Portuguese speakers. Only the Venezuelan study reported age-standardized overall dementia prevalence adjusted against the Venezuelan (7.42%; 95% CI: 6.38-8.46) and world standard (7.03%; 95% CI: 6.04-8.02) populations.

## DISCUSSION

In the elderly population aged 65 years and older studied in LA communities, the prevalence of FTD varied -depending on age- from 12 to 18 cases per 1000 persons. The FTD prevalence in LA was found to be higher in Brazilian than both Venezuelan and Peruvian populations, and had an intermediate values with respect to the published epidemiological studies worldwide.

The precise prevalence of FTD is unknown.^20^ Studies based on autopsy pathology estimate that FTD accounts for approximately 8% of patients with dementia.^14^ Most previous studies on the prevalence of FTD were based on data from hospitals^21^ and healthcare system.^22-27^ The reported prevalence in persons of any age referred to a memory clinic was 3.2%.^21^ The estimated community prevalence based on outpatient databases and case enrollment in health centers varied from 0.0176% to 0.035%, 0.002% to 0.031%, 0.078% to 0.156% and 0.054% to 0.135% in populations overall, aged 45 to 64, 65 to 74, and 75 years and over, respectively.^22-25,27^ However, recent door-to-door studies suggest that FTD is more common than previously thought.^13^ Only a few epidemiologic studies assessing overall FTD prevalence in the population are available. International door-todoor surveys have reported FTD prevalences from 0.3% to 3.5%13,^28-30^ in Western urban elderly populations. In Japanese rural communities, FTD prevalence ranges from 0 to 0.11%.^31,32^ Thus, the FTD prevalence in LA studies had values midway between those observed in Western European and Japanese oriental populations. However, Japanese populations studied were from rural areas, and therefore results may differ in Eastern urban populations.

This variation might indicate population differences (age structure, genetics, lifestyle, and environment), but could also be due to methodological variability (study and sample designs, diagnostic criteria) of these studies.

First, with respect to population differences, some studies focused on younger adult populations in order to assess early-onset dementia (EOD), which occurs before the age of 65. Among these, few door-to-door studies have specifically addressed FTD, a major cause of EOD.^13^ Accurate epidemiologic data are required for planning of health services because psychiatric and medical morbidity is particularly high in this group.^33^ However, FTD is not only an early-onset disorder, but is also frequent in advanced age in populations from LA.^34-38^

Second, the methodological aspects are important. Studies employ a variety of tests during the screening phase and different criteria for the diagnosis of dementia: [1] Diagnostic and Statistical Manual of Mental Disorders (version III, III-Revised, or IV); and/or [2] International Classification of Diseases, 10^th^ edition. Likewise, the criteria used for FTD diagnosis was not the same across all studies (Lund or Neary criteria).

Third, dementia as defined by DSM or similar criteria is not a requirement for the diagnosis of FTD. A frontal lobe syndrome (FLS) may be even more common as it often accompanies dementia disorders such as Alzheimer's disease and vascular dementia.^29^ Thus, FTD cases not fulfilling criteria for dementia would have been missed with such an approach in many studies, thereby underestimating the prevalence of FTD. Only two doorto- door studies have specifically addressed FTD^30,39^ and only one has reported on the prevalence of FTD by applying FTD criteria directly on unselected populations for dementia.^29^ Therefore, the real prevalence of FTD in Latin America may be underestimated.

These findings raise several questions which need to be elucidated. First, the prevalence of the disease needs to be better estimated in LA. Second, FTD as a worldwide cause of pre-senile dementia has been scarcely evaluated in community-dwelling population under 65 years old. Finally, the prevalence of subtypes of FTD and risk factors associated with this disorder are unknown in LA communities.

Results point to the need to recognize these patients with high accuracy during life in order to increase our understanding of this particularly devastating illness that uniquely compromises human functions while subjects are still productive. Thus, we need to reconsider its epidemiology and to rethink its impact on public policies. This is crucial to define the urgency of treatment approaches.

The prevalence of FTD in this region was found to be at a level midway between occidental and oriental studies. Highest and lowest dementia prevalence was observed in Venezuelan and Peruvian studies among the three countries studied. According to population-based studies conducted in individuals over 65 years of age in LA, the prevalence of FTD ranges from between 0.13% and 0.18%, ranking FTD as the second most common cause of degenerative dementia. Despite the magnitude of this problem, epidemiological information on FTD remains scarce in LA. Unfortunately, because of the relatively low prevalence, conventional "door-to-door studies" would be too costly and time consuming. Other additional methodological alternatives will be necessary for future FTD studies in LA.
